# Perioperative goal-directed minimally invasive hemodynamic therapy in surgery: a systematic review and meta-analysis

**DOI:** 10.1590/1806-9282.2026D726

**Published:** 2026-08-10

**Authors:** Antonio Silvinato, Idevaldo Floriano, Marques Bernardo

**Affiliations:** 1 Brazilian Medical Association, Evidence-Based Medicine – São Paulo (SP), Brazil.; 2 Universidade de São Paulo, Faculty of Medicine – São Paulo (SP), Brazil.

## INTRODUCTION

Perioperative hemodynamic management relies on the accurate estimation and replacement of fluid and blood losses. Traditionally, volume deficits are replaced using a 3:1 ratio (crystalloid volume to blood loss) to account for extravascular redistribution. Historically, this management has been guided by static parameters—such as mean arterial pressure, heart rate, and urinary output—to infer intravascular volume status.

In this context, maintaining euvolemia is a critical challenge and the central goal of perioperative care, as deviations from this balance are directly associated with increased morbidity. While hypovolemia leads to compensatory vasoconstriction, reduced global oxygen delivery (DO_2_), and impaired tissue perfusion, hypervolemia induces tissue edema and local inflammation. These latter effects compromise wound healing, increase the risk of surgical site infections, and predispose patients to anastomotic dehiscence.

Despite its clinical significance, a definitive consensus on optimal fluid management remains elusive, particularly for high-risk surgical patients with multiple comorbidities. To mitigate volume overload and optimize perfusion, goal-directed therapy (GDT) has emerged. Unlike traditional approaches, GDT utilizes dynamic physiological monitoring—such as cardiac output and stroke volume variation—to allow for the precise titration of crystalloids, colloids, or blood products.

Among available modalities, arterial waveform analysis has gained prominence. This method provides a continuous estimate of cardiac output by interpreting the interaction between left ventricular stroke volume, systemic vascular resistance, and vascular compliance.

Current evidence from meta-analyses suggests that individualized fluid management reduces postoperative complications in major surgery. However, the definitive superiority of these technological methods over traditional clinical judgment remains a subject of ongoing debate^1,2,3^. Therefore, it is essential to compare clinical outcomes—including complication rates and hospital length of stay—between patients receiving fluid management guided by arterial waveform analysis and those managed with traditional methods during high-risk surgery. The ESA Delphi Consensus defines major surgery multifactorially, considering procedural complexity, induced pathophysiological alterations, and expected clinical outcomes (high morbidity and mortality) rather than a simple list of procedures^4^.

## OBJECTIVE

To evaluate the benefits and harms of minimally invasive hemodynamic monitoring using uncalibrated pulse contour analysis (UC-PCA) compared to Standard of Care (SOC) in patients undergoing major or high-risk surgery, through a systematic review and meta-analysis of randomized controlled trials (RCTs).

## METHODS

This systematic review followed the Preferred Reporting Items for Systematic Reviews and Meta-analyses (PRISMA) guidelines^5^ and is supported by scientific evidence obtained through a systematic review of the published literature. The review was registered with PROSPERO (PROSPERO 2026—york.ac.uk) under registration number CRD420261333973.

### Eligibility criteria

The eligibility criteria address the specific elements required to answer the clinical question of this assessment (objective).

### Inclusion criteria

- Patients: Adults undergoing major or high-risk surgery, as defined by the European Society of Anesthesiology consensus.
- Intervention: Minimally invasive hemodynamic monitoring using real-time peripheral arterial pressure waveform analysis (pulse contour analysis), employing algorithms based on patient demographics and vascular characteristics to estimate stroke volume, without the need for external calibration (uncalibrated systems).
- Comparison: Standard care or conventional monitoring, guided by usual macro-hemodynamic parameters (such as Mean Arterial Pressure—MAP, Heart Rate—HR, Central Venous Pressure—CVP, and urine output), without the use of continuous pulse contour analysis technologies to guide hemodynamic therapy.
- Outcomes: Postoperative complications (morbidity and mortality); hospital length of stay; intensive care unit (ICU) length of stay; and duration of mechanical ventilation.
- Study design: Parallel-group randomized controlled trials.
- Language: No restrictions.
- Search period: No restrictions.
- Availability: Full text available.

Specificity regarding the monitoring technology was ensured by predefined eligibility criteria, restricting inclusion to uncalibrated pulse contour analysis systems based exclusively on a peripheral arterial line, without external calibration or the use of indicators.

### Exclusion criteria

The following were excluded: systematic reviews with or without meta-analysis; narrative reviews; observational studies and/or case series or studies with non-extractable data (absolute numbers and/or means); studies that used only non-invasive parameters based on pulse oximetry (such as the Pleth Variability Index—PVI), as they do not employ arterial pulse contour analysis for flow measurement.

### Evidence search

The literature search will be conducted in MEDLINE (via PubMed) using a combination of Medical Subject Headings (MeSH) and free-text terms. This will be supplemented by searches in EMBASE, the Cochrane Central Register of Controlled Trials (CENTRAL), and ClinicalTrials.gov. Search strategies were adapted to the specific syntax and indexing systems of each database; full details are provided in the Supplementary Table 1. Additionally, a manual search of the reference lists of included studies and relevant reviews will be performed, alongside a grey literature search to minimize the risk of omitting potentially eligible studies. The bibliographic search across these databases covers the period up to January 2026.

### Study selection and data extraction

Study selection was conducted in two stages. Initially, records identified in the databases underwent screening by title and abstract to identify potentially eligible studies based on pre-defined inclusion and exclusion criteria. Subsequently, the full texts of the selected studies were assessed to confirm eligibility.

Screening (titles, abstracts, and full texts) was performed independently and in a blinded manner by two reviewers experienced in systematic reviews (AS and IF). Disagreements were resolved by consensus or, if necessary, by consultation with a third reviewer (WMB).

Data extraction was performed independently by two reviewers (AS and IF) using a standardized, pilot-tested form. The following information was collected: lead author, year of publication, population characteristics, description of interventions and comparators, follow-up duration, and outcomes. For the outcomes of interest, data were extracted as absolute event numbers for dichotomous variables, and as measures of central tendency (means and/or medians) with appropriate measures of dispersion (standard deviations or 95%CI) for continuous variables.

### Risk of bias and quality of evidence

Two independent reviewers (AS, IF) assessed the risk of bias in the included studies using the Revised Cochrane Risk-of-Bias tool (RoB 2)^6^ This instrument evaluates five specific domains: (1) bias arising from the randomization process; (2) bias due to deviations from intended interventions; (3) bias due to missing outcome data; (4) bias in measurement of the outcome; and (5) bias in selection of the reported result. The overall risk of bias for each study was determined according to the tool’s criteria: a study was rated as “Low risk of bias” only if all domains were judged to be at low risk; “Some concerns” if at least one domain raised concerns but none were at high risk; and “High risk of bias” if at least one domain was judged to be at high risk or if multiple domains had “some concerns” in a way that substantially compromised confidence in the results.

Publication bias was assessed through visual inspection of funnel plots and Egger’s test^7^, contingent upon the inclusion of at least 10 studies. If fewer than 10 studies were available, publication bias was assessed qualitatively due to the low statistical power of quantitative tests in small samples^8^. A p-value of <0.05 was considered evidence of statistically significant publication bias. All statistical tests were two-tailed.

The certainty of the evidence was evaluated using the Grading of Recommendations Assessment, Development and Evaluation (GRADE)^9^ system, classifying the quality into four levels: high, moderate, low, or very low. The assessment, conducted by two reviewers, covered the domains of risk of bias, inconsistency, indirectness, imprecision, and publication bias. Analyses were performed using the GRADEpro GDT (Guideline Development Tool)^10^ software and are presented in Summary of Findings (SoF) tables (Supplementary Table 3).

### Data analysis and synthesis methods

Data will be analyzed according to the intention-to-treat (ITT) principle, prioritizing the most recent follow-up data from each clinical trial. For categorical outcomes, results will be expressed as Risk Difference (RD), calculated using the Mantel-Haenszel method. Should the RD reach statistical significance (95%CI), results will be supplemented by the Number Needed to Treat (NNT) or Number Needed to Harm (NNH).

For continuous variables, results will be expressed as Mean Difference (MD). In cases where different scales are used for the same outcome, the Standardized Mean Difference (SMD) will be employed. For studies that omit the Standard Deviation (SD), it will be estimated from the Standard Error (SE) or the 95%CI. Data presented as medians and intervals (interquartile range [IQR]/range) will be converted using the formulas by Luo et al.^11^ for the mean and Wan et al.^12^ for the SD.

Statistical synthesis will be performed using Review Manager (RevMan) software, version 5.4.^13^ Statistical heterogeneity will be quantified using the I^2^ index^14^. I^2^ values ≤50% will indicate the use of a fixed-effect model, while I^2^ values>50% will warrant a random-effects model, with heterogeneity further explored through sensitivity analysis.

Additional statistical analyses will be conducted using the “meta” package in the R statistical computing environment, version 4.5.1 (R Core Team, 2025)^15^.

### Evidence synthesis and conclusion

The evidence synthesis will report results directly from the analyses, accounting for benefit, harm, or lack of difference between UC-PCA and Standard of Care. Conclusions will be drawn based on evidence of at least moderate quality, the presence of an effect (either benefit or harm), and a favorable balance between benefits and risks in adult patients undergoing major or high-risk surgical procedures.

## RESULTS

In the search for evidence regarding the use of UC-PCA, 448 studies were retrieved from MEDLINE, 156 from EMBASE, 70 from CENTRAL, and 29 from ClinicalTrials.gov. No additional records were identified through manual searches or grey literature.

After the removal of duplicates and screening by title and abstract, 40 papers were selected for full-text assessment. Of these, 35 parallel-group randomized controlled trials (RCTs)^16,17,18,19,20,21,22,23,24,25,26,27,28,29,30,31,32,33,34,35,36,37,38,39,40,41,42,43,44,45,46,47,48,49,50^ met the eligibility criteria and were included to support the conclusions of this evaluation.

**Figure 1:**
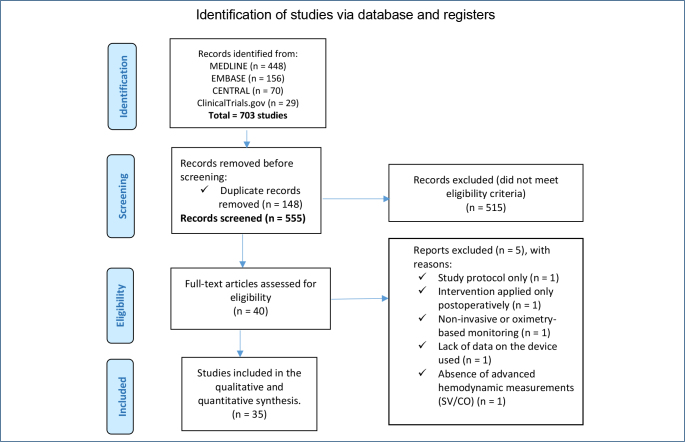
PRISMA 2020 flow diagram for study selection. Adapted from Page et al^5^.

Detailed reasons for the exclusion of the 5 studies after full-text review are listed in the “Excluded Studies and Reasons for Exclusion” section^51,52,53,54,55^. The flow diagram, illustrating the sequence from retrieval to the selection of evidence for this assessment, is presented in Figure 1.

The baseline characteristics and key details of each included trial are reported in Supplementary Table 2. These trials involved 4,903 participants (2,451 randomized to UC-PCA and 2,452 to the SOC group).

### Risk of bias in studies

The risk of bias assessment for each individual study, conducted using the RoB 2 tool^6^, is presented in Supplementary Figure 1. The analysis revealed that 1 study (2.9%) was at low risk of bias, 31 (88.6%) raised some concerns, and 3 (8.6%) were classified as high risk of bias. The most frequent sources of concern were deviations from intended interventions (33 studies), selection of the reported result (20 studies), measurement of the outcome (15 studies), randomization process (11 studies), and missing outcome data (9 studies).

### Outcomes

The certainty of evidence for each outcome, according to the GRADE system, is detailed in Supplementary Table 3.

#### 1. Mortality within 90 days

**Figure 2:**
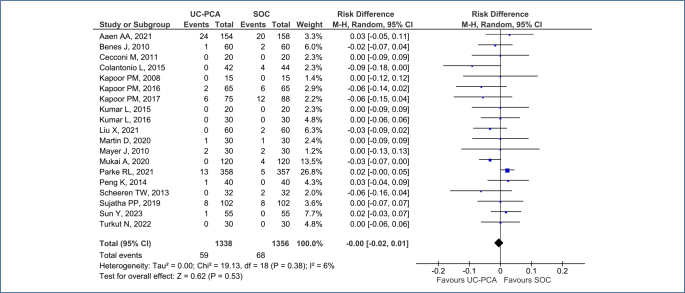
Forest plot of comparison: 1 UC-PCA versus SOC, outcome: 1.1 Mortality.

Nineteen studies, including a total of 2,694 participants, assessed the outcome mortality within 90 days, comparing UC-PCA with SOC. In this analysis, no difference in the risk of death was observed with UC-PCA compared with SOC (RD=-0.00; 95%CI -0.02 to 0.01; p=0.53; I^2^=6%), Figure 2.

Moderate certainty of evidence.

#### 2. Myocardial infarction

**Figure 3:**
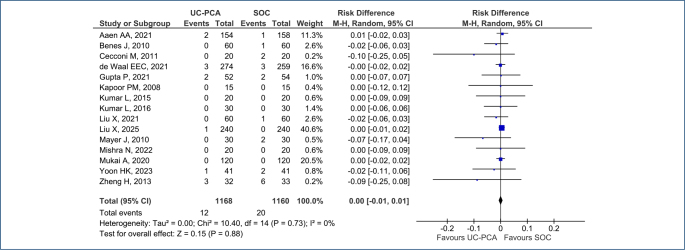
Forest plot of comparison: 1 UC-PCA versus SOC, outcome: 1.2 Myocardial infarction.

Fifteen studies, including a total of 2,328 participants, assessed the outcome myocardial infarction, comparing UC-PCA with SOC. In this analysis, no difference in the risk of myocardial infarction was observed between UC-PCA and SOC (RD=0.00; 95%CI -0.01 to 0.01; p=0.88; I^2^=0%), Figure 3.

Low certainty of evidence.

#### 3. Heart failure or pulmonary edema

Data on heart failure and pulmonary edema were combined into a composite outcome (acute heart failure) to mitigate variability in clinical reporting across studies.

**Figure 4:**
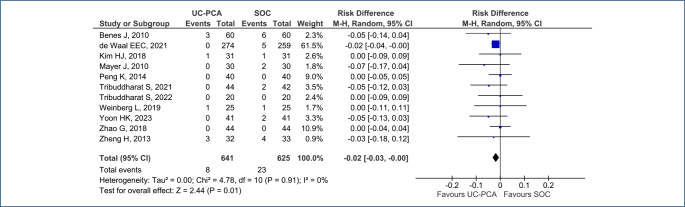
Forest plot of comparison: 1 UC-PCA versus SOC, outcome: 1.3 Acute Heart Failure or Pulmonary Edema. Note: RD values are rounded to two decimal places by the software; NNT was calculated using unrounded data.

Eleven studies, including a total of 1,266 participants, assessed this outcome by comparing UC-PCA with SOC. In this analysis, UC-PCA reduced the absolute risk of heart failure or pulmonary edema by 2% (RD=-0.02; 95%CI -0.03 to -0.00; p=0.01; I^2^=0%), Figure 4.

Although the upper limit of the confidence interval is visually rounded to -0.00 by the software, the estimated NNT^*^ was 40 (95%CI 24 to 125).

Low certainty of evidence.

#### 4. Acute kidney injury

**Figure 5:**
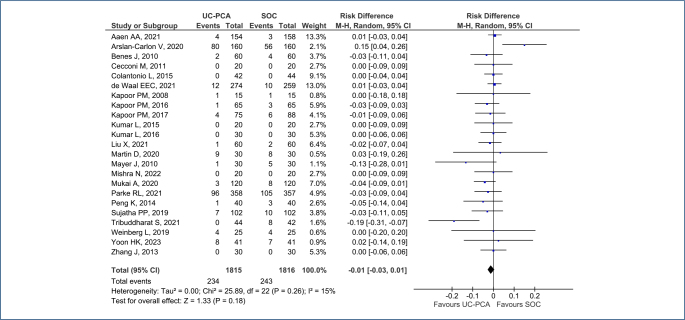
Forest plot of comparison: 1 UC-PCA versus SOC, outcome: 1.4 Acute kidney injury.

Twenty-three studies, including a total of 3,631 participants, assessed the outcome acute kidney injury, comparing UC-PCA with SOC. In this analysis, no difference in the risk of acute kidney injury was observed between UC-PCA and SOC (RD=-0.01; 95%CI -0.03 to 0.01; p=0.18; I^2^=15%), Figure 5.

Moderate certainty of evidence.

#### 5. Hypotension

**Figure 6:**
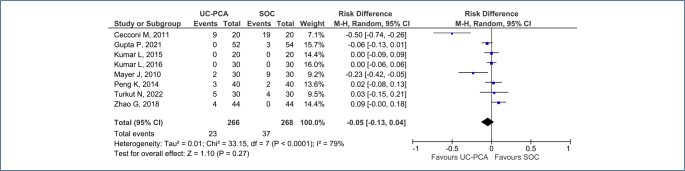
Forest plot of comparison: 1 UC-PCA versus SOC, outcome: 1.5 Hypotension.

Eight studies, including a total of 534 participants, assessed the occurrence of hypotension, comparing UC-PCA with SOC. In this analysis, no difference in the occurrence of hypotension was observed between UC-PCA and SOC (RD=-0.05; 95%CI -0.13 to 0.04; p=0.27; I^2^=79%), Figure 6.

Very low certainty of evidence.

#### 6. Hospital length of stay

**Figure 7:**
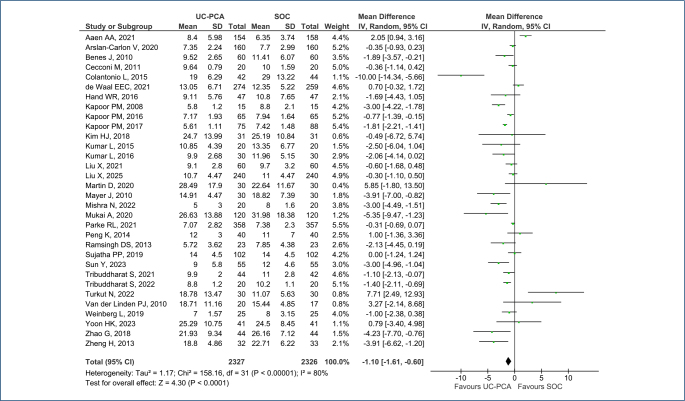
Forest plot of comparison: 1 UC-PCA versus SOC, outcome: 1.6 Hospital stay (days).

Thirty-two studies assessed hospital length of stay, comparing UC-PCA with SOC. The meta-analysis (Figure 7) showed a reduction in hospital length of stay in the intervention group, with a mean difference of -1.10 days (95%CI -1.61 to -0.60; p<0.0001).

Substantial heterogeneity was observed among the studies (I^2^=80.4%; 95%CI 73.0 to 85.8%), suggesting variability in methodological or population characteristics.

Low certainty of evidence.

#### 7. ICU length of stay

**Figure 8:**
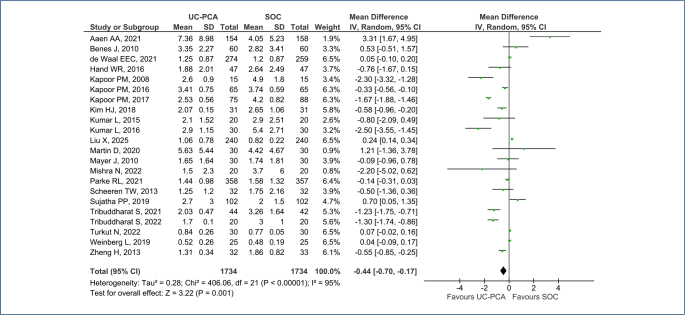
Forest plot of comparison: 1 UC-PCA versus SOC, outcome: 1.7 ICU stay (days).

Twenty-two studies assessed ICU length of stay, comparing UC-PCA with SOC. The meta-analysis (Figure 8) showed a reduction in ICU length of stay in the intervention group, with a mean difference of -0.44 days (95%CI -0.70 to -0.17; p=0.001).

Extremely high heterogeneity was observed among the studies (I^2^=95%; p<0.00001), indicating substantial variability in the results, which may be attributable to differences in study populations or unit protocols.

Very low certainty of evidence, mainly due to the inconsistency of results (high heterogeneity).

#### 8. Duration of mechanical ventilation

**Figure 9:**
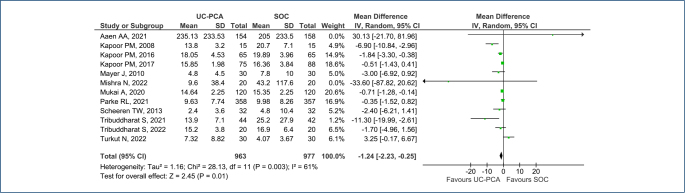
Forest plot of comparison: 1 UC-PCA versus SOC, outcome: 1.8 Duration of MV (hours).

Twelve studies assessed the duration of mechanical ventilation, comparing UC-PCA with SOC. The meta-analysis (Figure 9) showed a reduction in the duration of ventilatory support in the intervention group, with a mean difference of -1.24 h (95%CI -2.23 to -0.25; p=0.01).

Moderate to high heterogeneity was observed among the studies (I^2^=61%; p=0.003), suggesting clinical or methodological variability in ventilator weaning practices.

As with the length-of-stay outcomes, the certainty of evidence should be interpreted in light of the observed inconsistency across individual study results.

Low certainty of evidence.

### EVIDENCE SYNTHESIS

In patients undergoing major or high-risk surgery, minimally invasive hemodynamic monitoring using UC-PCA compared with SOC:

- Reduces hospital length of stay by a mean of 1.10 days (95%CI -1.61 to -0.60). Low-certainty evidence.
- Reduces ICU length of stay by a mean of 0.44 days (95%CI -0.70 to -0.17). Very low-certainty evidence.
- Reduces the duration of mechanical ventilation by a mean of 1.24 h (95%CI -2.23 to -0.25). Low-certainty evidence.
- Reduces the risk of heart failure or pulmonary edema by 2% (risk difference [RD] -0.02; 95%CI -0.03 to -0.00), corresponding to a NNT^*^ of 40 to prevent one event (95%CI 24 to 125). Low-certainty evidence.
- No difference was observed in the risk of death within 90 days (RD -0.00; 95%CI -0.02 to 0.01). Moderate-certainty evidence.
- No difference was observed in the risk of myocardial infarction (RD 0.00; 95%CI -0.01 to 0.01). Low-certainty evidence.
- No difference was observed in the risk of acute kidney injury (RD -0.01; 95%CI -0.03 to 0.01). Moderate-certainty evidence.
- No difference was observed in the occurrence of hypotension (RD -0.05; 95%CI -0.13 to 0.04). Very low-certainty evidence.

*Note: RD values are rounded to two decimal places by the software; NNT was calculated using unrounded data.

The GRADE certainty ratings for each outcome are presented in Supplementary Table 3.

## DISCUSSION

In this systematic review and meta-analysis, including 35 randomized controlled trials and 4,903 participants, we evaluated the effects of minimally invasive hemodynamic monitoring using UC-PCA compared with SOC in adult patients undergoing major or high-risk surgery, according to the consensus definition of the European Surgical Association. The findings show a consistent pattern: no detectable benefit in major clinical outcomes, accompanied by statistically significant but modest reductions in outcomes related to fluid overload and hospital resource utilization.

No difference was observed between UC-PCA-guided therapy and SOC for mortality up to 90 days, myocardial infarction, or acute kidney injury. The mortality analysis showed low heterogeneity and moderate certainty of evidence, supporting the robustness of the estimated absence of effect. These findings are consistent with the contemporary literature on goal-directed hemodynamic therapy, in which perioperative interventions rarely demonstrate an effect on mortality when applied to heterogeneous surgical populations with variable baseline risk. From a pathophysiological perspective, although UC-PCA enables dynamic optimization of preload and, in some protocols, cardiac index, outcomes such as myocardial infarction and acute kidney injury are multifactorial events influenced by organ reserve, inflammatory response, surgical stress, transfusion practices, and overall anesthetic management. Consequently, optimization of fluid responsiveness alone may be insufficient to substantially modify these outcomes.

A reduction was observed in the composite outcome of acute heart failure or pulmonary edema, with no heterogeneity across studies. Although the absolute risk reduction was small, the finding is physiologically plausible, as protocols based on stroke volume variation aim to prevent both hypovolemia and fluid overload, thereby reducing the likelihood of pulmonary congestion and postoperative cardiac dysfunction. However, the certainty of evidence was low, mainly due to methodological limitations and the small absolute number of events.

UC-PCA was also associated with reductions in hospital length of stay, ICU length of stay, and duration of mechanical ventilation, suggesting that individualized hemodynamic optimization may facilitate postoperative recovery and reduce the use of critical care resources. These findings should nevertheless be interpreted cautiously. Substantial statistical heterogeneity, particularly for ICU length of stay, indicates variability across surgical populations, hemodynamic protocols, and institutional discharge criteria. In addition, the absolute magnitude of effect—especially for ventilation duration—was small, which may limit reproducibility across different clinical settings.

The methodological quality of the included trials is an important consideration. Most studies were classified as having “some concerns” according to the Cochrane RoB 2 tool, mainly due to lack of blinding and potential deviations from intended interventions—limitations that are largely inherent to trials involving hemodynamic monitoring. Only one study was judged to have an overall low risk of bias.^32^ In addition, evidence of publication bias was identified for the outcome of myocardial infarction, which may have influenced the estimated effect size. These considerations justified downgrading the certainty of evidence for several outcomes according to the GRADE framework.

Compared with previous meta-analyses by Chong et al.^3^ and Giglio et al.^1^, which reported reductions in complications with goal-directed therapies, the present study differs by restricting the analysis exclusively to uncalibrated systems based on peripheral arterial waveform analysis. This restriction increases the internal validity of the evaluation for this specific technology but may partly explain the smaller magnitude of effect observed compared with strategies that use calibrated systems or more invasive techniques to estimate absolute cardiac output.

### Strengths and limitations of this review

This review has several strengths. It included a large number of randomized controlled trials and participants, and the analysis was restricted to uncalibrated pulse contour analysis systems, increasing the internal validity of the evaluation for this specific technology. In addition, the methodological quality of the evidence was systematically assessed using the Cochrane RoB 2 tool and the GRADE framework. However, some limitations should be acknowledged. The included trials showed heterogeneity in surgical populations, hemodynamic protocols, and outcome definitions, which may have contributed to the variability observed in length-of-stay outcomes. Furthermore, most studies presented some concerns regarding risk of bias, mainly related to lack of blinding, and indications of publication bias were identified for at least one outcome. These factors reduce the overall certainty of evidence and should be considered when interpreting the results.

From a clinical perspective, the findings suggest that UC-PCA is safe and may reduce complications related to fluid overload while also being associated with improvements in indicators of hospital efficiency.

However, no high-certainty evidence currently supports its universal adoption as a standalone standard of care for the purpose of reducing mortality or major organ events. Implementation should therefore consider institutional context, patient risk profile, cost-effectiveness, and integration with multimodal perioperative recovery strategies. It is plausible that benefits may be greater in higher-risk hemodynamic subgroups or in settings with greater variability in conventional management—hypotheses that warrant further investigation in pragmatically designed multicenter trials.

Overall, UC-PCA-based hemodynamic monitoring appears to provide small but consistent benefits in intermediate outcomes and hospital resource utilization, without detectable impact on mortality or major cardiovascular and renal events. The coexistence of statistical significance with low to moderate certainty of evidence warrants cautious interpretation, and high-quality trials are still needed to clarify the role of this technology in defining perioperative standards of care.

## CONCLUSION

Minimally invasive hemodynamic monitoring using UC-PCA, compared with standard care, was associated with reductions in hospital length of stay (1.10 days), intensive care unit length of stay (0.44 days), and duration of mechanical ventilation (1.24 h). In addition, a 2% absolute reduction in the risk of acute heart failure or pulmonary edema was observed, corresponding to a NNT of 40 (95%CI 24–125).

No differences between the interventions were observed for mortality up to 90 days, myocardial infarction, acute kidney injury, or the occurrence of hypotension.

According to the GRADE framework, the certainty of evidence ranged from low to very low, reflecting methodological limitations in the primary studies—including risks of bias related to limited blinding in several trials—and substantial statistical inconsistency in the length-of-stay outcomes. Although UC-PCA may represent a promising strategy for optimizing perioperative hemodynamic management and potentially reducing complications related to fluid overload, its clinical implementation should consider the local care context. Further well-designed randomized trials are needed to increase confidence in the estimated effects.

## Data Availability

The datasets generated and/or analyzed during the current study are available from the corresponding author upon reasonable request.
