# Supplementary material for: Perioperative goal-directed minimally invasive hemodynamic therapy in surgery: a systematic review and meta-analysis
Source: Rev Assoc Med Bras (1992). 2026 Aug 10;72(6):e2026D726. doi: 10.1590/1806-9282.2026D726 (PMC13459721; doi:10.1590/1806-9282.2026D726)
Supplement: Supplementary file 1 [file 1806-9282-ramb-72-6-e2026D726-supp1.docx]

**Supplementary Figure 1.** Risk of bias assessment (Traffic light plot). D: domain.

**Supplementary Table 1.** Databases searched and search strategy.

| **Database** | **Search strategy** | **Articles** |
| --- | --- | --- |
| MEDLINE/PubMed | {“Early Goal-Directed Therapy”[Mesh] OR “Goals”[Mesh] OR “minimally invasive hemodynamic monitoring” OR “cardiac output monitor” OR (“minimally invasive” AND “goal directed”) OR “goal-directed” OR “stroke volume” OR “Monitoring, Intraoperative”[Mesh]} AND “Fluid Therapy”[Mesh] AND Random* | 448 |
|  |  |  |
| EMBASE | {(“early goal-directed therapy”/exp OR “early goal-directed therapy” OR “goals”/exp OR “goals” OR “minimally invasive hemodynamic monitoring” OR “cardiac output monitor”/exp OR “cardiac output monitor” OR (“minimally invasive” AND “goal directed”) OR “goaldirected” OR “stroke volume”/exp OR “stroke volume” OR “monitoring, intraoperative”/exp OR “monitoring, intraoperative”) AND “fluid therapy” AND [randomized controlled trial]/lim} AND [embase]/lim NOT ([embase]/lim AND [medline]/lim) | 156 |
|  |  |  |
| CENTRAL (Cochrane Central Register of Controlled Trials)/Cochrane Library | (“pulse contour” OR “pulse contour analysis” OR “minimally invasive hemodynamic monitoring” OR “cardiac output monitoring”) AND (“goal-directed” OR “goal directed” OR “goal-directed fluid therapy”) AND (surgery OR surgical OR intraoperative OR perioperative) | 70 |
|  |  |  |
| ClinicalTrials.gov | (“minimally invasive hemodynamic monitoring” OR “hemodynamic monitoring” OR “hemodynamic monitor” OR “haemodynamic monitoring” OR “device minimally invasive hemodynamic monitoring”) AND (“goal directed” OR “Goal-directed” OR “Stroke Volume” OR “Monitoring, Intraoperative” OR “Fluid Therapy”) \| Interventional studies | 29 |

**Supplementary Table 2.** Characteristics of included studies.

| Study | N (I/C)* | Population/type of surgery | Hemodynamic protocol (GDT) | Main reported outcomes |
| --- | --- | --- | --- | --- |
| Liu et al.^16^ | 240/240 | 18–65 years old; elective supratentorial high-grade glioma resection. | GDT (SVV): Bolus 3 mL/kg HES if SVV >15% for 5 min; vasopressors if MAP <80% basal. | Myocardial infarction, hospital stay (days), ICU stay (days). |
| Sun et al.^17^ | 55/55 | Elective major abdominal surgery. | GDT (SVV/CI): Bolus 250 mL if SVV >12% for 5 min; inotropes if CI <2.5 L/min/m²; vasopressors if MAP <65 mmHg. | Mortality, hospital stay (days). |
| Yoon et al.^18^ | 41/41 | Aged >20 years; open radical cystectomy; ASA I–III. | GDT (SVI/CI): Bolus 250 mL colloid to optimize SVI (plateau); dobutamine if CI <2.5 L/min/m²; norepinephrine if MAP <65 mmHg. | Myocardial infarction, acute heart failure/pulmonary edema, acute kidney injury, hospital/ICU stay. |
| Turkut et al.^19^ | 30/30 | 18–80 years; ASA I–III; head and neck surgery. | GDT (SVV): Bolus 250 mL colloid if SVV ≥10% for 5 min; vasopressors if MAP <65 mmHg. | Mortality, hypotension, hospital/ICU stay, duration of MV (hours). |
| Mishra et al.^20^ | 20/20 | 18–65 years; craniotomy for large supratentorial tumors. | GDT (SVV): Bolus 3 mL/kg crystalloid if SVV >13% for 5 min; vasopressors if MAP <20% basal. | Myocardial infarction, acute kidney injury, hospital/ICU stay, duration of MV. |
| Tribuddharat et al.^21^ | 20/20 | Adults; CABG with cardiopulmonary bypass; ASA II and IV. | GDT (SVI/CI): Bolus 250 mL colloid to optimize SVI (increase ≥10%); inotropes if CI <2.5 L/min/m². | Acute heart failure/pulmonary edema, hospital/ICU stay, duration of MV. |
| Aaen et al.^22^ | 154/158 | Adults; gastrointestinal obstruction or perforation. | GDT (SV): Bolus 200–250 mL colloid to optimize SV (increase ≥10%); reassessment every 15 min. | Mortality, myocardial infarction, acute kidney injury, hospital/ICU stay, duration of MV. |
| Gupta et al.^23^ | 52/54 | 18–70 years; head and neck malignancy; composite resection with free tissue transfer. | GDT (SVV): Bolus 250 mL colloid if SVV ≥13% for 10 min; vasopressors if MAP <65 mmHg. | Myocardial infarction, hypotension. |
| Liu et al.^24^ | 60/60 | >65 years old; elective open gastrointestinal surgery; ASA 2–4. | GDT (SVV/CI): Bolus 200 mL crystalloid if SVV >12%; dobutamine if CI <2.5; pre-op carbohydrate loading. | Mortality, myocardial infarction, acute kidney injury, hospital stay. |
| Parke et al.^25^ | 358/357 | ≥16 years; elective cardiac surgery; EuroSCORE II ≥0.9. | GDT (SVV): Bolus 250–500 mL if SVV >13%; vasopressors if MAP out of target. | Mortality, acute kidney injury, hospital/ICU stay, duration of MV. |
| Tribuddharat et al.^26^ | 44/42 | ≥18 years; elective CABG with bypass; ASA II–IV. | GDT (SVI/CI): Bolus 250 mL colloid if SVI increase ≥10%; dobutamine if CI <2.5. | Acute heart failure/pulmonary edema, acute kidney injury, hospital/ICU stay, duration of MV. |
| Waal et al.^27^ | 274/259 | ≥18 years; elective high-risk abdominal surgery. | GDT (CI/SVV): Bolus 500 mL if SVV >12%; dobutamine if CI below age-specific target. | Myocardial infarction, acute heart failure/pulmonary edema, acute kidney injury, hospital/ICU stay. |
| Mukai et al.^28^ | 120/120 | >20 years; elective open transthoracic or thoraco-laparoscopic esophagectomy. | GDT (CI/SVV): Bolus 500 mL if SVV >12%; dobutamine if CI below age-specific target. | Mortality, myocardial infarction, acute kidney injury, hospital/ICU stay, duration of MV. |
| Arslan-Carlon et al.^29^ | 160/160 | Adults; open radical cystectomy. | GDT (SVV/SVI): Bolus 250 mL if SVV >13% or SVI drop >10%; vasopressors if MAP <60 mmHg. | Acute kidney injury, hospital stay. |
| Martin et al.^30^ | 30/30 | 18–80 years with liver cirrhosis; liver transplantation. | GDT (SV): Bolus 250 mL colloid if SV drop >10%; target maximum SV. | Mortality, acute kidney injury, hospital/ICU stay. |
| Sujatha et al.^31^ | 102/102 | 20–70 years; ASA I-II; elective open major bowel surgery. | GDT (SVV/SV): Bolus 250 mL crystalloid if SVV >12%; repeat if SV increase ≥10%. | Mortality, acute kidney injury, hospital/ICU stay. |
| Weinberg et al.^32^ | 25/25 | >18 years; elective major liver resection. | GDT (SV): Bolus 250 mL colloid if SV drop >10% for 5 min; basal fluid restriction (1 mL/kg/h). | Acute heart failure/pulmonary edema, acute kidney injury, hospital/ICU stay. |
| Kim et al.^33^ | 31/31 | 20–80 years; elective free flap reconstruction for head and neck cancer. | GDT (SV): Bolus 250 mL colloid if SV drop >10%; target maximum SV. | Acute heart failure/pulmonary edema, hospital/ICU stay. |
| Zhao et al.^34^ | 44/44 | >60 years; elective radical correction of gastrointestinal cancer. | GDT (SVV/CI): Bolus 200 mL colloid if SVV >12%; dobutamine if CI <2.5 L/min/m². | Acute heart failure/pulmonary edema, hospital stay. |
| Kapoor et al.^35^ | 75/88 | >18 years; elective CABG; EuroSCORE ≥3. | GDT (CI/SVV/ScvO_2_): Bolus 250 mL if SVV >12%; inotropes if CI <2.5 or ScvO_2_ <70%. | Mortality, acute kidney injury, hospital/ICU stay, duration of MV. |
| Hand et al.^36^ | 47/47 | Adults; head and neck free tissue transfer and reconstruction. | GDT (SV): Bolus 250 mL colloid if SV drop >10%; target maximum SV. | Mortality, acute kidney injury, hospital/ICU stay. |
| Kapoor et al.^37^ | 65/65 | Adults; EuroSCORE ≥3; CABG on cardiopulmonary bypass. | GDT (CI/SVV/ScvO_2_): Bolus 250 mL if SVV >12%; inotropes if CI <2.5 or ScvO_2_ <70%. | Mortality, acute kidney injury, hospital/ICU stay, duration of MV. |
| Kumar et al.^38^ | 30/30 | Adults; ASA I–II; major abdominal surgery. | GDT (SVV): Bolus 250 mL colloid if SVV >10%; maintain SVV <10%. | Mortality, myocardial infarction, acute kidney injury, hypotension, hospital/ICU stay. |
| Kumar et al.^39^ | 20/20 | Adults; ASA II–III; major abdominal surgery. | GDT (CI/O_2_ER): Bolus 250 mL if CI <2.5; dobutamine if O2ER >30% or low CI. | Mortality, myocardial infarction, acute kidney injury, hypotension, hospital/ICU stay. |
| Colantonio et al.^40^ | 42/44 | ASA II–III; peritoneal carcinomatosis; peritonectomy and HIPEC. | GDT (SVI): Bolus 250 mL colloid if SVI drops >10%; target maximum SVI. | Mortality, acute kidney injury, hospital stay. |
| Peng et al.^41^ | 40/40 | Adults; elective major orthopedic surgery. | GDT (SVV): Bolus 4 mL/kg if SVV >10% (supine) or >14% (prone); target low SVV. | Mortality, acute heart failure/pulmonary edema, acute kidney injury, hypotension, hospital stay. |
| Scheeren et al.^42^ | 32/32 | ≥18 years; ASA III or IV; major abdominal surgery or radical cystectomy. | GDT (SVV/SV): Maintain SVV <10% and SV plateau (increase <10% after bolus). | Mortality, ICU stay (days), duration of MV. |
| Ramsingh et al.^43^ | 23/23 | Adults; major abdominal non-vascular surgery. | GDT (SVV): Bolus 250 mL colloid if SVV ≥12%; maintain SVV <12%. | Hospital stay (days). |
| Zhang et al.^44^ | 40/40 | 18–60 years; ASA I–II; thoracoscopic lobectomy. | GDT (SVV/CI): Maintain SVV at 10% (±1%); bolus 200 mL and dobutamine if CI <2.5. | Acute kidney injury. |
| Zheng et al.^45^ | 32/33 | Elderly; coronary heart disease; gastrointestinal surgery. | GDT (SVV/SVI): Bolus 200 mL colloid if SVV >12%; target maximum SVI. | Myocardial infarction, acute heart failure/pulmonary edema, hospital/ICU stay. |
| Cecconi et al.^46^ | 20/20 | Adults; elective total hip arthroplasty. | GDT (SV/DO₂I): Maintain SV plateau. If DO_2_I <600 mL/min/m² after plateau, start dobutamine 3 µg/kg/min. | Mortality, myocardial infarction, acute kidney injury, hypotension, hospital stay. |
| Mayer et al.^47^ | 30/30 | Adults; ASA III; open major abdominal surgery. | GDT (CI): Bolus 250 mL colloid if CI <3.0 L/min/m²; repeat until CI plateau. | Mortality, myocardial infarction, acute heart failure/pulmonary edema, acute kidney injury, hypotension, hospital/ICU stay, MV. |
| Linden et al.^48^ | 20/17 | Adults; ASA II–III; peripheral arterial bypass grafting. | GDT (CI): Maintain CI >2.5 L/min/m²; bolus 250 mL colloid if CI below target. | Hospital stay (days). |
| Benes et al.^49^ | 60/60 | High-risk adults; ASA ≥3; major abdominal surgery. | GDT (SVV): Bolus 3 mL/kg colloid if SVV ≥10%; maintain SVV <10%. | Mortality, myocardial infarction, acute heart failure/pulmonary edema, acute kidney injury, hospital/ICU stay. |
| Kapoor et al.^50^ | 15/15 | Adults; EuroSCORE ≥3; CABG with cardiopulmonary bypass. | GDT (CI/SvO_2_): Bolus and inotropes to maintain CI >2.8 L/min/m² and SvO_2_ >65%. | Mortality, myocardial infarction, acute kidney injury, hospital/ICU stay, duration of MV. |

ASA: American Society of Anesthesiologists; CI: cardiac index; DO_2_I: Oxygen Delivery Index; GDT: goal-directed therapy; HES: Hydroxyethyl Starch; HIPEC: hyperthermic intraperitoneal chemotherapy; MAP: mean arterial pressure; MV: mechanical ventilation; O_2_ER: Oxygen Extraction Ratio; ScvO_2_: Central Venous Oxygen Saturation; SV: stroke volume; SVI: Stroke Volume Index; SVV: stroke volume variation; N (I/C): number of participants in Intervention group/control group; ICU: intensive care unit.

**Supplementary Table 3.** Levels of evidence—GRADE system

Summary of findings:

UC-PCA compared to SOC—benefit/harm

Patient or population: patients undergoing major or high-risk surgery.

Context: Efficacy and safety

Intervention: UC-PCA

Comparison: SOC

| **Outcomes** | **Anticipated absolute effects* (95%CI)** | | **№ of participants (studies)** | **Certainty of the evidence (GRADE)** |
| --- | --- | --- | --- | --- |
|  | **Risk with SOC** | **Risk with UC-PCA** |  |  |
| Mortality | 50 per 1,000 | **50.147 per 1,000** (40.147 to 70.147) | 2,694 (19 RCTs) | ⨁⨁⨁◯  Moderate^a^ |
| Myocardial infarction | 17 per 1,000 | **17.241 per 1,000** (7.241 to 27.241) | 2,328 (15 RCTs) | ⨁⨁◯◯  Low^a,b^ |
| Acute Heart Failure or Pulmonary Edema | 37 per 1,000 | **56.8 per 1,000** (36.8 to 66.8) | 1,266 (11 RCTs) | ⨁⨁◯◯  Low^a,c^ |
| Acute kidney injury | 134 per 1,000 | **143.811 per 1,000** (123.811 to 163.811) | 3,631 (23 RCTs) | ⨁⨁⨁◯  Moderate^a^ |
| Hypotension | 138 per 1,000 | **188.06 per 1,000** (98.06 to 268.06) | 534 (8 RCTs) | ⨁◯◯◯  Very low^a,d,e,f^ |
| Hospital stay (days) | The mean hospital stay (days) was **0** | MD **1.1 lower** (1.61 lower to 0.6 lower) | 4,653 (32 RCTs) | ⨁⨁◯◯  Low^a,g^ |
| ICU stay (days) | The mean ICU stay (days) was **0** | MD **0.44 lower** (0.7 lower to 0.17 lower) | 3,468 (22 RCTs) | ⨁◯◯◯  Very low^a,h^ |
| Duration of MV (hours) | The mean duration of MV (hours) was **0** | MD **1.24 lower** (2.23 lower to 0.25 lower) | 1,940 (12 RCTs) | ⨁⨁◯◯  Low^a,i^ |

^a^Methodological limitations in the primary studies, specifically the lack of blinding of participants and personnel (inherent to the intervention) and uncertainties regarding the selection of the reported result, as evidenced by the Traffic Light Plot. ^b^Downgraded for the myocardial infarction outcome due to statistical significance in Egger’s test (p=0.0031). ^c^Downgraded by one level due to the low absolute number of events (n=31) and the width of the Risk Difference confidence interval (95%CI -0.03 to -0.00), which demonstrates a borderline benefit very close to nullity. Additionally, the NNT confidence interval (24–125) reflects uncertainty regarding the true magnitude of the clinical effect. ^d^Downgraded by one level due to high statistical heterogeneity (I^2^=79%), suggesting significant variability in the intervention effects across different centers or hypotension management protocols. ^e^Downgraded due to insufficient sample size to reach the optimal information size and a confidence interval that crosses the line of nullity, precluding confirmation of the direction of the effect. ^f^Downgraded due to suspected publication bias, suggested by visual asymmetry in the funnel plot and the possibility that small studies with negative results remained unpublished. ^g^Downgraded by one level due to high statistical heterogeneity (I^2^=80.4%), indicating that the intervention effect varies significantly across different hospital settings. ^h^Downgraded by two levels due to extreme heterogeneity (I^2^=95%). This variability suggests that the difference of -0.44 days may not be reproducible across all clinical scenarios. ^i^Downgraded by one level due to moderate-to-high heterogeneity (I^2^=61%), suggesting that ventilator weaning protocols varied between studies. SOC: Standard of Care; UC-PCA: uncalibrated pulse contour analysis; RCT: randomized controlled trial; MD: mean difference; MV: mechanical ventilation.
